# Ulcer healing and mechanism(s) of action involved in the gastroprotective activity of fractions obtained from *Syngonanthus arthrotrichus* and *Syngonanthus bisulcatus*

**DOI:** 10.1186/s12906-015-0923-x

**Published:** 2015-10-29

**Authors:** Leônia Maria Batista, Gedson Rodrigues De Morais Lima, Ana Beatriz Albino De Almeida, Luciana De Pietro Magri, Tamara Regina Calvo, Anderson Luiz Ferreira, Cláudia Helena Pellizzon, Clélia Akiko Hiruma-Lima, Wagner Vilegas, Paulo Takeo Sano, Alba Regina Monteiro Souza Brito

**Affiliations:** Departamento de Ciências Farmacêuticas, Centro de Ciências da Saúde, Laboratório de Farmacologia do Trato Gastrintestinal, Universidade Federal da Paraíba (UFPB), João Pessoa, PB Brazil; Departamento de Fisiologia e Biofísica, Instituto de Biologia, Universidade Estadual de Campinas (UNICAMP), Campinas, SP Brazil; Departamento de Fisiologia, Instituto de Biociências, Universidade Estadual Paulista, Botucatu, SP Brazil; Departamento de Morfologia, Instituto de Biociências, Universidade Estadual Paulista, Botucatu, SP Brazil; Departamento de Botânica, Instituto de Biociências, Universidade de São Paulo, São Paulo, Brazil

**Keywords:** Eriocaulaceae, *Syngonanthus arthrotrichus*, *Syngonanthus bisulcatus*, Flavonoid, Healing, Gastroprotective activity

## Abstract

**Background:**

*Syngonanthus arthrotrichus* and *Syngonanthus bisulcatus*, currently known for *Comanthera aciphylla* (Bong.) L.R.Parra & Giul*.* and *Comanthera bisulcata* (Koern.) L.R. Parra & Giul, popularly known in Brazil as “*sempre-vivas*,” are plants from the family Eriocaulaceae. They are found in the states of Minas Gerais and Bahia. The species are known to be rich in flavonoids to which their gastroprotective activity has been attributed. In this research, experimental protocols were performed to elucidate the associated mechanisms of action.

**Methods:**

The activity was evaluated using induced gastric ulcer models (acetic acid and ethanol-induced gastric lesions in NEM or L-NAME pre-treated mice, and by ischemia/reperfusion). Antioxidant enzymes, serum somatostatin, and gastrin were also evaluated.

**Results:**

In chronic gastric ulcers, a single daily oral dose of *Sa*-FRF or *Sb*-FRF (100 mg/kg body wt.) for 14 consecutive days accelerated ulcer healing to an extent similar to that seen with an equal dose of cimetidine. The pre-treatment of mice with NEM (N-ethylmaleimide) or L-NAME (N-nitro-L-arginine) abolished the protective activity of *Sa*-FRF, *Sa*-FDF, Sb-FDF and *Sb*-FRF or *Sa*-FRF and *Sb*-FRF, respectively, which indicates that antioxidant compounds and nitric oxide synthase activity are involved in the gastroprotective. *Sa*-FRF and *Sb*-FRF (100 mg/kg p.o) protected the gastric mucosa against ulceration that was induced by ischemia/reperfusion (72 and 76 %, respectively). It also decreased lipid peroxidation and restored total thiols in the gastric wall of mice that had been treated with ethanol. When administered to rats submitted to ethanol-induced gastric lesions, *Sa*-FRF and *Sb*-FRF (100 mg/kg, p.o.) increased the somatostatin serum levels, while the gastrin serum levels were proportionally decreased.

**Conclusions:**

The results indicate significant healing effects and gastroprotective activity for the *Sa*-FRF and *Sb*-FRF, which probably involves the participation of SH groups, nitric oxide (NO), the antioxidant system, somatostatin, and gastrin. All are integral parts of the gastrointestinal mucosa’s cytoprotective mechanisms against aggressive factors.

## Background

Gastric ulcer is a disease that affects many people around the world, and its progression is attributed to an imbalance between aggressive factors (acid, pepsin, *Helicobacter pylori,* stress, alcohol, and continued use of non-steroidal anti-inflammatory drugs), and protective factors (mucus, bicarbonate, prostaglandin, blood flow, the antioxidant system, sulfhydryl compounds, nitric oxide and cell proliferation) [1–3].

Gastric ulcer is a common disease with multiple etiologies. Factors such as smoking, stress, an improper diet, and gastro-protective deficiencies are closely related to the development of gastric mucosa ulceration. Bacterial infection with *Helicobacter pylori* has also been highlighted as an important predisposing factor for developing gastric ulcers [4].

Thru the years, a great store of knowledge has been acquired concerning the development of gastric ulcer, and a broad spectrum of drugs for its treatment such as antacids, proton pump inhibitors, anticholinergics, and histamine receptor antagonists [5], has developed. Nevertheless, the existing therapies commonly have adverse effects (hypersensitivity, arrhythmia, impotence, gynecomastia and hematopoietic changes), and are expensive. This has stimulated continued research for new therapeutic alternatives. At this point, we insert medicinal plants for their various advantages such as greater availability, more effective protection, lower cost, and lower toxicity [6, 7].

The early search in the area of medicinal plant in the treatment of peptic ulcers opened the discovery of the first drug effective against peptic ulcer; carbenoxolone from *Glycyrrhiza glabra* [8–10], and licorice root fluid extract were used to treat stomach ulcers in patients had not improved with conventional medication. The glycyrrhizin of licorice was found to stop two enzymes that break down prostaglandin E [11]. Effectiveness of other plant resources as cabbage in improving peptic ulcers have been reported [12–14].

Among the medicinal plants presented as candidates for the treatment of gastric ulcer we note those belonging to the Eriocaulaceae family. The family consists of more than 10 genera, being roughly 1200 species. Though it has a pan tropical distribution, most species occur in near tropical regions, such as in the mountains of Venezuela, or in Brazil [15–17]. Eriocaulaceae is the dominant herbal family of the Cipó Mountain range in the state of Minas Gerais, Brazil. We focused on species belonging to the genus *Syngonanthus* (*Comanthera*)*, S. arthrotrichus, and S. bisulcatus.* These species are currently known as *Comanthera aciphylla and Comanthera bisulcata* [18]*,* respectively, and popularly known as “sempre-vivas-mini-saia” and “sempre-vivas-chapadeira".

The phytochemical screening of the extracts of *Syngonanthus bisulcatus* (*Comanthera bisulcata *(Koern.) L.R. Parra & Giul) and *Syngonanthus artrotrichus* (*Comanthera aciphylla* (Bong.) L.R.Parra & Giul) performed by Batista [19] showed that the main compounds present in the extracts were flavonoids and phenolic compounds.

The chromatographic study of the flavonoid rich fractions obtained from the scapes of the species *S. bisulcatus* and *S. arthrotricus* was performed as described by Batista [19]. The species *Syngonanthus bisulcatus* presented major compounds such as luteolin and glycosylated derivatives of luteolin confirming the previous studies described by Agrawal [20] and Harbone [21].

Studies performed by Coelho [22] showed in this fraction also the presence of isovitexin, lutonarine and 5, 6, 3’, 4’-tetrahidroxi-7-O-D-glucopiranosilflavone. In the flavonoid rich fractions of *Syngonanthus arthrotrichus* was possible to isolate the compound luteolin. In studies previously described by Agrawal [20] and Rinaldi [23] were also identified luteolina, apigenin and luteolin-6-C-β-D-glucopiranoside.

Research has shown that medicinal plants promote anti-inflammatory, antioxidant, and gastroprotective effects [24–29]. The gastroprotective activity promoted by flavonoids has been demonstrated in a literature review published by Mota et al. [30]. Studies performed by our group have shown the gastroprotective activity of plant species such as *S. arthrotrichus* [31]*, S. bisulcatus* [32], and *S. macrolepsis* [33] all of which were collected in the state of Minas Gerais, Brazil.

Considering the above, we aimed to explore gastroprotective effects promoted by fractions obtained from *S. arthrotrichus* and *S. bisulcatus*, using varied induced gastric ulcer models, to thus evaluate the mechanisms of action involved.

## Methods

### Animals

The experimental protocols were approved by the Committee for Ethics in Animal Experimentation (CEEA/UNICAMP) with register number 502–1, in 2002. Male Swiss albino mice (30–40 g) or male Wistar albino rats (180–250 g), from the Central Animal House of the State University of Campinas (CEMIB/UNICAMP) were used. The animals were fed a certified Nuvilab CR-diet, and in addition, had free access to water under fixed conditions of illumination (12/12 h light/dark cycle), humidity (60 ± 1.0 %), and a temperature of (21,5° ± 1.0). Fasting was used prior to all assays, because standard drugs were administered orally (by gavage), or by the intra-duodenal route using a 0.9 % saline solution (10 mL/kg) as the vehicle (negative control). The animals were kept in cages with raised wide mesh floors to prevent coprophagy.

### Drugs

The drugs and reagents were prepared immediately before use. The following drugs were used: cimetidine (Sigma Chemical Co, USA), lansoprazole (Aché, Brazil), absolute ethanol (Merck, Germany), hydrochloric acid (Merck, Germany), Nω-Nitro-L-arginine methyl ester hydrochloride (Sigma Chemical Co, USA), and N-Ethylmaleimide (Sigma Chemical Co, USA). All drugs and fractions were administered orally (gavage), or intraduodenally.

### Plant material

Scapes of *S. arthrotrichus,* and *S. bisulcatus* were collected in 2002 at Cipó Mountain, Diamantina City, in the state Minas Gerais, Brazil. *S. arthrotrichus* was authenticated by Dr. Ana Maria Giulietti, and *S. bisulcatus* was authenticated by Dr. Paulo Takeo Sano. A voucher of each specimen (n° 2122 and 2137, respectively) was deposited in the Herbarium of the Department of Botany, at the Institute of Biosciences, USP.

Scapes (500 g) of *S. arthrotrichus* and *S. bisulcatus* collected in the Serra do Cipó, Minas Gerais, were dried in an oven at 60 °C for 4 d and then powdered. The resulting material was macerated sequentially at room temperature in methylene chloride, EEOH and 70 % EEOH for one week with each solvent. The extracts were filtered and concentrated under vacuum*.* The EEOH and 70 % EEOH extracts were analyzed by TLC on silica gel plates using n- BuOH/HOAc/H2O (6:1:2, v/v/v). The TLC spots were detected using UV light and NP/PEG reagent which yields yellow or orange spots characteristic of flavonoids. Since these extracts contained material with similar retention factors (Rf ), they were combined and weighed. A sample (3.5 g) of the ethanolic extract was dissolved in 10 ml of MeOH and fractionated on a Sephadex LH-20 CC column (10033 cm). The extract was eluted in MeOH at a flow rate of 0.5 ml/min and 3 ml fractions were collected. The fractions were combined based on their migration in the TLC system described above. Fractions 1–22 were deficient in flavonoids, fractions 23–47 were intermediate fractions and fractions 58–64 were rich in flavonoids and were administered at a dose of 100 mg/Kg.

### Healing in acetic acid-induced gastric lesion

The experiment was performed according to the method described by Takagi et al. [34], with some modifications. After fasting for 24 h, and under anesthesia, a laparotomy was performed on all animals through a midline epigastric incision. After exposing the stomach, a 30 % (v/v) acetic acid solution (0.05 mL) was injected into the subserosal layer in the glandular part of the anterior wall. The abdomen was then closed and all the animals were fed normally. Two days after the acetic acid indution of gastric lesion, treatments were initiated with oral administrations of *Sa*-FRF, *Sa*-FDF; *Sb*-FRF, *Sb*-FDF; cimetidine (100 mg/kg); or 0.9 % saline solution at 10 mL/kg. All treatments were administered orally once a day for 14 consecutive days. On the day after the last drug administration, (and after fasting for 12 h), the rats were sacrificed, and their stomachs then removed. The macroscopic ulcer lesion area (ULA) of the internal and external borders in mm^2^ were determined as described in the method.

### Ethanol-induced gastric lesions in NEM pre-treated mice

The mice were divided into groups (*n* = 5–7 animals), and were fasted for 24 h. They were then treated intraperitoneally with NEM (N-ethylmaleimide) at 10 mg/kg, or 0.9 % saline solution at 10 mL/kg. At thirty minutes, the groups received an oral dose of either 0.9 % saline solution, *Sa*-FRF, *Sa*-FDF, *Sb*-FRF or *Sb*-FDF. After 60 min, all groups were orally treated with 0.2 mL of HCl/ethanol to induce gastric-ulcer [35]. One hour after the administration of HCl/ethanol the animals were euthanized by cervical dislocation and their stomachs were excised. Gastric damage was determined using the ulcerative index (UI) as described by Szelenyi and Thiemer [36].

### Ethanol-induced gastric mucosal lesion in L-NAME-pre-treated mice

The mice were fasted for 24 hours and divided into 3 groups (*n* = 5–7), according to pre-treatments: one group received 0.9 % saline solution (10 mL/kg, s.c.), and two groups received Nω-L-arginine methyl ester (L-NAME), an oxide synthase blocking agent, at 70 mg/kg (s.c.). Thirty minutes after administration, the groups were orally treated with either 0.9 % saline solution, *Sa*-FRF, *Sa*-FDF, *Sb*-FRF or *Sb*-FDF. Sixty minutes later, all groups were treated orally with 0.2 mL HCl/ethanol to induce gastric ulcer [37]. One hour later, the animals were euthanized by cervical dislocation, the stomachs were excised, and gastric damage was determined as previously described.

### Gastric lesion induced by ischemia/reperfusion

Ischemia/reperfusion damage was produced in the rats by a method proposed by Ueda et al. [38]. The rats were fasted for 24 hours and divided (according to pre-treatment) into four groups (*n* = 5–7): normal (no pre-treatment and neither ischemia nor reperfusion), sham (no pre-treatment), 0.9 % saline (10 mL/kg), and *Sa*-FRF or *Sb*-FRF. After 15 min, the rats were anaesthetized by an intramuscular injection of Ketamine (50 mg/kg) and Xylazine (10 mg/kg). The left side of the abdomen was shaved, and an incision was made. Briefly, the celiac artery was dissected, free of excess fat, and clamped for 30 minutes (ischemia phase), using a micro-bulldog clamp. Re-oxygenation was then allowed by removal of the clamp for 60 min (reperfusion phase). The macroscopic ulcer lesion area were determined. 

### Antioxidant activity

To assess the effects of the *Sa*-FRF and *Sb*-FRF on the antioxidative system, we assayed lipid peroxidation, and the levels of sulfhydryls (GSH), in the glandular stomach mucosa in those rats with ischemia/reperfusion gastric lesions.

### Lipid Peroxidation (LPO)

The total thiobarbituric acid-reactive substances (TBARS) level in gastric tissue, (an index of malonyldialdehyde (MDA) production), was determined according to the method developed by Ohkawa et al. [39]. Gastric segments were cut into small pieces and then homogenized in an ice-cold phosphate buffer (50 mM, pH 7.4), to give a 10 % homogenate. The homogenate of the glandular portion of stomach was diluted in 0.15 M KCl (ratio 1:10). Then to 0.5 mL of this homogenate were added 0.2 mL of dodecyl sulfate (8.1 %), 1.5 ml of acetic acid 20 %, (adjusted with an NaOH solution to pH 3.5), 1.5 ml of thiobarbituric acid (0.8 % w/v), and 0.3 mL of distilled water. Subsequently, the mixture was heated in a water bath (bain-marie) with the thermostat set at 95 °C for 1 hour. After this, the samples were cooled and added to 1 mL of distilled water, and 5 mL of an n-buthanol + pyridine (15:1, v/v) mixture, and shaken in a vortex for 1 min, and then centrifuged at 1400 G for 10 minutes. Finally the absorbance was read at 532 nm. TEPP (1,1,3,3-tetraethoxypropane) diluted in ethanol was used as the standard. The results were expressed as picomoles of the substances reacting with the thiobarbituric acid (TBARS), per mg of protein (nmol TBARS . mg protein^−1^).

### Total thiol groups in stomach homogenate

The total thiols in the gastric tissue were determined according to the Faure and Lafond [40] method with modifications. Briefly, 500 μL of 0.25 mM Tris, and 20 mM EDTA solution (pH 8.2), was added to an aliquot (100 mL) of the homogenate (in a phosphate 10 mM buffer, pH 7.8) obtained from the stomach scraping. The absorbance (A1) of the final solution was determined by spectrophotometer at 412 nm. Then, 10 μL of 5-5’-dithio-bis (2-nitrobenzoic acid) (DTNB) 10 mM (diluted in methanol) was added to the mixture. After 15 min, we took a second reading (A2). DTNB diluted in buffer was used as blank (B). To calculate the sulfhydryl groups concentration we used the following equation: (A2 – A1-B) × 1,57 mM. The results were expressed in nmol TBARS/mg protein × 10^3^.

### Serum somatostatin and gastrin

To perform this protocol, gastric lesions were induced by oral administration of absolute ethanol 1 mL [41]. Thirty minutes before lesion induction, the animals received an oral dose of the 0.9 % saline solution, lansoprazole (30 mg/kg), *Sa*-FRF or *Sb*-FRF. One hour after ethanol administration; blood was collected by the abdominal aorta and placed into tubes containing EDTA. The sample was centrifuged at 3000 G for 15 minutes, and the plasma obtained from each sample was stored at −20 ° C until serum somatostatin and gastrin determinations.

### Somatostatin

The somatostatin dosage in the plasma of the animals was performed according to methodology described by Arimura et al. [42], using a radioimmunoassay “kit” EURIA-Somatostatin (RB-306, Eurodiagnóstica). Residual radioactivity was determined in a gamma scintillation counter (Beckman, model G5500), with a time of 2–4 minutes.

### Gastrin

Plasma gastrin determination was performed according to the method described by Slingerland et al. [43], using a radioimmunoassay “kit” (CIS bio International - GASK-PR). Residual radioactivity was determined in a gamma scintillation counter (Beckman, Model G5500) for 1 minute.

### Statistical analysis

Results were expressed as mean ± S.D. or mean ± S.E.M. Statistical significance between groups was determined by one-way analysis of variance (ANOVA) followed by Dunnett’s tests, with *p* < 0.05 considered significant. The statistical software program utilized was GraphPad Prism® version 4 (U.S.A., 2003).

## Results

### Healing in acetic acid-induced gastric lesion

The effects of the *S. arthrotrichus* and *S. bisulcatus* fractions in the acetic acid-induced gastric lesion model are shown in Table 1. The results show that the *Sa*-FRF (42 ± 1,6*), *Sb*-FRF (49 ± 4,3*), and cimetidine (22 ± 2,5**) significantly reduced the ULA, when compared to the saline group (75 ± 8,6). Yet, *Sa*-FDF and *Sb*-FDF did not promote significant protection. These results suggest that *Sa*-FRF and *Sb*-FRF demonstrated healing of the lesions induced by acetic acid.

**Table 1:** Effects of fractions obtained from *Syngonanthus arthrotrichus* and *Syngonanthus bisulcatus* on healing in acetic acid-induced gastric lesion

Gastric lesion models	Treatment (p.o.)	Dose (mg/kg)	ALU (mm^2^)	Inhibition (%)
Acetic acid	Saline	-	75 ± 8,6	-
	Cimetidine	100	22 ± 2,5**	71
	Sa-FRF	100	42 ± 1,6*	44
	Sa-FDF	100	65 ± 7,0	13
	Sb-FRF	100	49 ± 4,3*	35
	Sb-FDF	100	67 ± 25	11

ANOVA followed by Dunnett’s test.*p < 0.05, **p < 0.01. Data are presented as mean ± S.D (*n* = 5-7)

### Ethanol-induced gastric lesions in NEM pre-treated rats

For ethanol-induced gastric lesions in the NEM pre-treated rats model, we observed that when the groups were pre-treated with NEM (a sulfhydryl group blocker), and treated with either *Sa*-FRF (53 ± 11**), *Sa*-FDF (56 ± 13**), *Sb*-FRF (56 ± 11*), *Sb*-FDF (69 ± 17*), or saline (76 ± 21**, 83 ± 14*) an increase of the UI occurred when compared to the controls (27 ± 8) (Table 2). The results demonstrate that the sulfhydryl compounds pathway is involved in the gastroprotective effect promoted by the fractions studied.

**Table 2:** Effects of fractions obtained from *Syngonanthus arthrotrichus* and *Syngonanthus bisulcatus* on gastric lesions induced by ethanol in rats pretreated with NEM

Pretreatment	Treatment (p.o.)	Dose (mg/kg)	UI (mm)	Increase Lesion (%)
Saline	Saline	10	27 ± 8	-
NEM (10 mg/Kg)	Saline	10	76 ± 21**	181
NEM (10 mg/Kg)	Sa-FRF	100	53 ± 11**	96
NEM (10 mg/Kg)	Sa-FDF	100	56 ± 13**	107
Saline	Saline	10	27 ± 8	-
NEM (10 mg/Kg)	Saline	10	83 ± 14*	207
NEM (10 mg/Kg)	Sb-FRF	100	56 ± 11*	107
NEM (10 mg/Kg)	Sb-FDF	100	69 ± 17*	155

ANOVA followed by Dunnett’s test.* *p* < 0.05, ** *p* < 0.01. Data are presented as mean ± S.D (*n* = 5-7)

### Ethanol-induced gastric mucosal lesion in L-NAME-pre-treated rats

We observed that groups pretreated with L-NAME and subsequently treated orally with the *Sa*-FRF (39 ± 5,4*) or *Sb*-FRF (32 ± 6,3*) displayed an increase of the UI when compared to their respective saline control groups (17 ± 5,5, 16 ± 5,7) (Table 3). *Sa*-FDF and *Sb*-FDF did not promote significant UI changes. The results suggest that NO is related to the gastroprotective effects promoted by *Sa*-FRF and *Sb*-FRF*.*

**Table 3:** Effects of fractions obtained from *Syngonanthus arthrotrichus* and *Syngonanthus bisulcatus* on gastric lesions induced by ethanol in rats pretreated with L-NAME

Pretreatment	Treatment (p.o.)	Dose (mg/kg)	UI (mm)	Increase lesion (%)
Saline	Saline	10	17 ± 5,5	-
L-NAME (70 mg/Kg)	Saline	10	59 ± 15*	247
L-NAME (70 mg/Kg)	Sa-FRF	100	39 ± 5,4*	129
L-NAME (70 mg/Kg)	Sa-FDF		22 ± 1,9	29
Saline	Saline	10	16 ± 5,7	-
L-NAME (70 mg/Kg)	Saline	10	60 ± 14**	275
L-NAME (70 mg/Kg)	Sb-FRF	100	32 ± 6,3*	100
L-NAME (70 mg/Kg)	Sb-FDF	100	20 ± 3,5	25

ANOVA followed by Dunnett’s test.*p < 0.05, **p < 0.01. Data are presented as mean ± S.D (*n* = 5-7)

### Gastric lesion induced by ischemia/reperfusion

Pretreatment with the *Sa*-FRF or *Sb*-FRF significantly protected the gastric mucosa against gastric lesion induced by ischemia/reperfusion (52 ± 15* and 41 ± 25*, respectively), when compared with the saline group (181 ± 26) (Table 4). These results suggest that *Sa*-FRF and *Sb*-FRF protect the gastric mucosa from ischemia/reperfusion-related injuries.

**Table 4:** Effects of the flavonoid-rich fraction (FRF) from *Syngonanthus arthrotrichus* and *Syngonanthus bisulcatus* on gastric lesions induced by ischemia and reperfusion

Gastric lesion models	Treatment (p.o.)	Dose (mg/kg)	ALU (mm^2^)	Inhibition (%)
Ischemia and Reperfusion	Normal	-	-	-
	Saline	10	181 ± 26	0
	Sham	-	156 ± 29	14
	Sa-FRF	100	52 ± 15*	72
	Sb-FRF	100	41 ± 25*	76

ANOVA followed by Dunnett’s test.*p < 0.05, **p < 0.01. Data are presented as mean ± S.D (*n* = 5-7)

### Antioxidant activity

#### Lipid Peroxidation (LPO)

We observed a significant decrease in lipid peroxidation for the treated rats with *Sa*-FRF (47 ± 8,1**), and *Sb*-FRF (51 ± 6,6**) when compared with the saline group (115 ± 8,9). Hence, substances present in either fraction may well be associated with antioxidant activity (Table 5).

**Table 5:** Effects of the flavonoid-rich fraction (FRF) from *Syngonanthus arthrotrichus* and *Syngonanthus bisulcatus* on the activities of antioxidative enzymes in the gastric mucosa of rats with ethanol–induced lesions

Treatments	Dose (mg/kg)	Lipid peroxidation (TBARS/mg protein x 10^−3^)	Inibition (%)	Total thiols ( μmol/mg protein)	Increase (%)
Saline	-	115 ± 8,9	-	13 ± 2,7	-
Sham	10	98 ± 6,7*	15	15 ± 5,9	15
Normal	-	49 ± 8,3**	57	31 ± 7,2**	138
Sa-FRF	100	47 ± 8,1**	59	24 ± 4,2**	61
Sb-FRF	100	51 ± 6,6**	56	21 ± 6,7*	84

ANOVA followed by Dunnett’s test.* *p* < 0.05, ** *p* < 0.01. Data are presented as mean ± S.D (*n* = 5-7)

#### Total thiol groups in stomach homogenate

The levels of total thiols increased in the rats treated with flavonoid rich fractions obtained from *S. arthrotrichus* (24 ± 4,2**), and *S. bisulcatus* (21 ± 6,7*) when compared with the saline group (13 ± 2,7). Decreasing GSH was prevented by treatment with the studied plant samples (Table 5).

#### Serum Somatostatin and Gastrin

According to the results for the somatostatin model, we observe that the *Sa-*FRF (77 ± 17*), the *Sb*-FRF (82 ± 8,2*), and lanzoprazole (88 ± 22*) significantly increased serum somatostatin levels when compared with the saline group (20 ± 7,5). On the other hand we also observed that animals treated with *Sa*-FRF (59 ± 20*), and *Sb*-FRF (76 ± 9,3*) showed significant reductions in serum gastrin levels when compared to the control group (349 ± 22). This suggests that the gastroprotective effects promoted by both *S. arthrotrichus,* and *S. bisulcatus* are associated with inhibition of gastric acid secretion (Table 6).

**Table 6:** Effects of the flavonoid-rich fraction (FRF) from *Syngonanthus arthrotrichus* and *Syngonanthus bisulcatus* on the serum somatostatine and gastrin

Treatment (p.o.)	Somatostatin (pmol/L)	Gastrin (μU/mL)
Sham	21 ± 1,5	334 ± 15
Saline	20 ± 7,5	349 ± 22
Lansoprazole	88 ± 22*	47 ± 7,1*
Sa-FRF	77 ± 17*	59 ± 20*
Sb-FRF	82 ± 8,2*	76 ± 9,3*

ANOVA followed by Dunnett’s test.*p < 0.05, **p < 0.01. Data are presented as mean ± S.D (*n* = 5-7)

## Discussion

In the present study we investigated the healing activity, and possible mechanisms of action involved in the gastroprotective effects promoted by the *S. arthrotrichus* and *S. bisulcatus* species*.* Previous studies conducted by Batista et al. [31, 32] have demonstrated that significant gastroprotective effects are promoted by ethanolic extracts, and fractions (FRF, and FDF at doses of 100 mg/ kg), in acute gastric ulcer induced-models. The best results were obtained with the flavonoid fractions, and this encouraged our research group to continue the study, focusing on the *Sa*-FRF, *Sa*-FDF, *Sb*-FRF and *Sb*-FDF.

Healing and gastroprotective effects promoted by medicinal plants have been widely investigated in a number of studies [44–46]. The acetic acid induced gastric ulcer model in rats has been used to evaluate healing activity for many years. Acetic acid-induced lesions most resemble the ulcers occurring in man in terms of location, severity, and chronicity as well as in the physiological processes related to healing [47]. It is known that the healing process involves cell proliferation and migration, restoration of the gland architecture, angiogenesis, and matrix deposition [48–50]. Histamine H_2_-receptor antagonists and proton-pump inhibitors generally accelerate the healing of gastric ulcers through their potent and long-lasting antisecretory actions [51].

The results obtained in our experiments confirmed that animals treated with *Sa*-FRF or *Sb*-FRF enjoyed significant cure rates when compared to the control group. The results suggest healing promotion. The good results obtained with *Sa*-FRF and *Sb*-FRF encouraged the authors to continue the research with experimental protocols assessing the contribution of sulfhydryl groups to the gastroprotective effect.

Non-protein sulfhydryl compounds (NP-SH) play an important role, scavenging free radicals, as well as acting against toxic substances that are either ingested or produced in the intestines. These groups act by binding to free radicals or by forming disulfide bonds in the gastric mucus, thus preventing cleavage [52, 53]. L-NAME pretreatment of the *Sa*-FRF, *Sa*-FDF, *Sb*-FRF or *Sb*-FDF animals increased the UI when compared to the controls. These results suggest that the protective effects of analyzed fractions of the species studied are dependent on sulfhydryl compounds.

The release of nitric oxide (NO) causes vasodilation of sub-mucosal arterioles, and increases blood flow. This increase in blood flow improves the buffering capacity of the gastric acid entering the lamina propria as well as helping to dilute and remove other toxins that cross the epithelium [54]. Furthermore, nitric oxide participates in gastric defense mechanisms by regulating acid, alkaline, and mucus secretions [55].

The results showed that the UI significantly increased in groups pretreated with L-NAME compared to the control group. However, the UI increases in animals pretreated with the FRFs (of both species), were lower than those obtained in the absence of these fractions, which indicates that some gastric mucosa protection is afforded by the FRFs. When comparing pre-treatments with FDFs, of both species, we found that they blocked completely any worsening of L-NAME-induced lesions. This suggests that the mechanism of action (where the FRFs of both species failed to protect the gastric mucosa) is dependent on NO. The effects exerted by the FDFs were not dependent NO.

Gastric ischemia and reperfusion relates to important indices of morbidity and mortality, in episodes of hemorrhagic shock, bleeding ulcers, and diseases of the gastrointestinal tract. The available treatments for ischemia and gastric reperfusion related injuries are still underperformers; and the search for sources of new therapeutics has become important [56]. Our next step was to evaluate gastroprotective effects against gastric ulcers as induced by ischemia and reperfusion.

Ischemia and reperfusion cause the release of factors responsible for tissue damage, such as reactive oxygen species, and chemotaxis of inflammatory cells, with the consequent release of pro-inflammatory mediators [57–60]. Ischemia breaks the gastric mucous barrier, and increases back-diffusion of acid, thus predisposing the gastric mucosa to injury. In turn, after reperfusion the formation of reactive oxygen species from the xanthine oxidase system occurs, which leads to lipid peroxidation which along with gastric secretion results in cell injury and death [61, 62]. The results suggest that the *Sa*-FRF and *Sb*-FRF display gastroprotective activity at the dose evaluated. This protection could reflect antioxidant and anti-inflammatory activities promoted by the plant samples studied.

Continuing the study, we performed tests to confirm pro-antioxidant activity. For this we used homogenates of the rat stomachs (having been first submitted to ischemia and reperfusion), with the aim of determining total thiols, and lipid peroxidation.

Experimental evidence indicates that depletion of cellular GSH levels leads to accumulation of reactive oxygen species, which may be responsible for aggravating the gastric ulceration process [63]. Increases in the levels of reactive oxygen species are directly related to lipid peroxidation. This can be observed in the formation of products such as MDA [64, 65].

Our results show that animals treated with *Sa-*FRF or *Sb*-FRF, and then subjected to ischemia and reperfusion were able to prevent lipid peroxidation, as well as reverse reductions in thiol levels for the injured gastric tissue. Based on these results, antioxidant activity is inferred for the plant samples. At this stage of the study, the authors judged the mechanisms of action to be only partially understood, with certain effects related to gastric acid secretion needing to be further clarified. Therefore experiments to evaluate the impact of plant samples on serum levels of gastrin and somatostatin were performed.

Somatostatin, produced by D cells in the mucosa of the stomach and pancreas is a regulator of stomach acid, and gastrin [66] releases. In ulcerated animals, plasma somatostatin levels decrease [67]. When the plasma levels of somatostatin were determined in the rats pretreated with *S. bisulcatus* and *S. artrotrichus* FRF at 100 mg/kg, increases in these hormone levels were observed in relation to the control group. This suggested our hypothesis that the protective mechanism of the FRFs involves inhibition of gastric acid secretion.

Gastrin, produced by G cells of the stomach and the duodenum, in turn, stimulates parietal cell cholecystokinin-β receptors through elevated intracellular calcium levels, and increases acid secretion [68, 69]. It has been observed that in ulcerated animals, plasma gastrin levels increase [67]. When we investigated the possible hormonal role of FRFs (for pre-treated animals) in gastrin secretion, we observed that there was a significant reduction in gastrin plasma levels. These data confirmed the involvement of the *Syngonanthus* fractions in gastric acid secretion mechanisms, and explain part of the gastroprotective activity of the plant samples studied.

## Conclusion

In conclusion, our results suggest that both *S. bisulcatus* and *S. artrotrichus* FRFs were responsible for wound healing in acetic acid induced ulcers. The anti-ulcer activity is related to decreased acid secretion, the presence of sulfhydryl compounds, to nitric oxide which causes reductions of somatostatin and gastrin, to reduced lipid peroxidation, and to an increase in thiol groups.

## References

[1] Li WF, Hao DJ, Fan T, Huang HM, Yao H, Niu X (2014). Protective effect of chelerythrine against ethanol-induced gastric ulcer in mice. Chem Biol Interact.

[2] Maity P, Biswas K, Roy S, Banerjee RK, Bandyopadhyay U (2003). Smoking and the pathogenesis of gastroduodenal ulcer-recent mechanism update. Mol Cell Biochem.

[3] Wallace JL, Granger DN (1996). The cellular and molecular basis of gastric mucosal defense. Faseb J.

[4] Mózsik G, Jávor T (1988). A biochemical and pharmacological approach to the genesis of ulcer disease. I. A model study of ethanol-induced injury to gastric mucosa in rats. Dig Dis Sci.

[5] Malfertheiner P, Chan FKL, McColl KEL (2009). Peptic ulcer disease. Lancet.

[6] Lin PC, Chang CH, Hsu PI, Tseng PL, Huang YB (2010). The efficacy and safety of proton pump inhibitors vs histamine-2 receptor antagonists for stress ulcer bleeding prophylaxis among critical care patients: a meta-analysis. Crit Care Med.

[7] Bansal VK, Goel RK (2012). Gastroprotective effect of *Acacia nilotica* young seedless pod extract: role of polyphenolic constituents. Asian Pac J Trop Med.

[8] Henmann FD (1970). Inhibition of peptic activity by carbenoxolone and glycerrhetinic acid. Gut.

[9] Baron JH (1977). Effect of carbenoxolone sodium on human gastric acid secretion. Gut.

[10] Ali AM, Al-Alousi L, Sae-lem HA (2005). Licorice: A possible anti-inflammatory and Anti-ulcer drug. AAPS Pharm Sci Tech.

[11] Shibata S (2000). A drug over the millennia: pharmacognosy, chemistry, and pharmacology of licorice [review]. Yakugaku Zasshi.

[12] Adami E, Marzzi-Uberti E, Turba C (1964). Pharmacological research on gefarnate, a new synthetic isoprenoid with antiulcer action. Arch Int Pharmacol Therap.

[13] Best R, Lewis DA, Nasser N (1984). The antiulcerogenic activity of unripe plantain banana (Musa spp.). Brit J Pharmacol.

[14] Goel RK, Gupta S, Shankar R, Sanyal AK (1986). Anti-ulcerative effect of Banana powder (*Musa sapientum* var. paradisiacal) and its effect on mucosal resistance. J Ethnopharmacol.

[15] Giulietti AM, Pirani JR, Heyer WR, Vanzolini PE (1988). Patterns of geographic distribution of some plant species from the Espinhaço Range, Minas Gerais and Bahia, Brazil. Proceedings of a Workshop on Neotropical Distribution Patterns.

[16] Giulietti AM, Hensold N (1990). Padrões de distribuição geográfica dos gêneros de Eriocaulaceae. Acta Bot Bras.

[17] Lazzari LRP. Redelimitacão e revisão de *Syngonanthus* Sect. Eulepis (Bong. ex Koern.) Ruhland – Eriocaulaceae. *PhD Thesis.* Universidade de São Paulo, 2000.

[18] Parra LR, Giulietti AM, Andrade MJG, Van Den Berg C (2010). Reestablishment and new circumscription of *Comanthera* (Eriocaulaceae). Taxon.

[19] Batista LM (2003). Atividade Antiulcerogênica de Extratos e Frações obtidas dos escapos das espécies *Syngonanthus bisulcatus* Rul. e *Syngonanthus arthrotrichus* Silveira em modelos animais. *PhD Thesis.*.

[20] Agrawal PK (1989). Carbon-13 NMR of flavonoids.

[21] Harbone JB (1996). The Flavonoids advances in research since 1986.

[22] Coelho RG (2000). Estudo químico de Sempre-Vivas brasileiras: *Syngonanthus bisulcatus* (Eriocaulaceae).

[23] Rinaldi V (2000). Estudo químico de plantas da família Eriocaulaceae. 2000. Monografia(Conclusão de curso).

[24] De Morais Lima GR, de Albuquerque Montenegro C, de Almeida CL, de Athayde-Filho PF, Barbosa-Filho JM, Batista LM (2011). Database survey of anti-inflammatory plants in South America: A review. Int J Mol Sci.

[25] De Morais Lima GR, de Sales IR, Caldas Filho MR, de Jesus NZ, de Sousa FH, Barbosa-Filho JM (2012). Bioactivities of the genus *Combretum* (Combretaceae): a review. Molecules.

[26] Lima GRM, Montenegro CA, Falcão HS, Jesus NZT, Cabral AG, Gomes IF (2013). Gastroprotective activity of the ethanolic extract and hexane phase of *Combretum duarteanum* Cambess. (Combretaceae). J Nat Med.

[27] Falcão HS, Maia GL, Bonamin F, Kushima H, Moraes TM, Hiruma Lima CA (2013). Gastroprotective mechanisms of the chloroform and ethyl acetate phases of *Praxelis clematidea* (Griseb.) R.M.King & H.Robinson (Asteraceae). J Nat Med.

[28] De Sousa Falcão H, Leite JA, Barbosa-Filho JM, de Athayde-Filho PF, de Oliveira Chaves MC, Moura MD (2008). Gastric and duodenal antiulcer activity of alkaloids: a review. Molecules.

[29] Jesus NZ, Falcão HS, Lima GR, Caldas Filho MR, Sales IR, Gomes IF (2013). *Hyptis suaveolens* (L.) Poit (Lamiaceae), a medicinal plant protects the stomach against several gastric ulcer models. J Ethnopharmacol.

[30] Mota KS, Dias GE, Pinto ME, Luiz-Ferreira A, Souza-Brito AR, Hiruma-Lima CA (2009). Flavonoids with gastroprotective activity. Molecules.

[31] Batista LM, de Almeida AB, de Pietro ML, Toma W, Calvo TR, Vilegas W (2004). Gastric antiulcer activity of *Syngonanthus arthrotrichus* Silveira. Biol Pharm Bull.

[32] Batista LM, de Almeida ABA, de Morais Lima R, de Sousa Falcão H, Ferreira AL, de Pietro Magri L (2013). Gastroprotective Effect of the Ethanolic Extract and Fractions obtained from *Syngonanthus bisulcatus* Rul. Rec Nat Prod.

[33] Batista LM, de Almeida AB, Lima GR, Falcão Hde S, Magri Lde P, Luiz-Ferreira A (2014). Gastroprotective effects (in rodents) of a flavonoid rich fraction obtained from *Syngonanthus macrolepsis*. J Pharm Pharmacol.

[34] Takagi K, Okabe S, Saziki R (1969). A new method for the production of chronic gastric ulcer in rats and the effect of several drugs on its healing. Jap J Pharmac.

[35] Matsuda H, Li Y, Yoshikawa M (1999). Gastroprotections of escins Ia, Ib, Iia, and Iib on ethanol-induced gastric mucosal lesions in rats. Eur J Pharmacol.

[36] Szelenyi I, Thiemer K (1978). Distention ulcer as a model for testing of drugs for ulcerogenic side effects. Arch Toxicol.

[37] Sikirić P, Seiwerth S, Grabarević Z, Rucman R, Petek M, Jagić V (1997). The influence of a novel pentadecapeptide, BPC 157, on NG-nitro-L-arginine methylester and L-arginine effect on stomach mucosa integrity and blood pressure. Eur J Pharmacol.

[38] Ueda S, Yoshikawa T, Takahashi S, Ichikawa H, Yasuda M, Oyamada H (1989). Role of free radicals and lipid peroxidation in gastric mucosal injury induced by ischemia-reperfusion in rats. Scand J Gastroenterol.

[39] Ohkawa H, Ohishi N, Yagi K (1979). Assay for lipid peroxidation in animal tissues by thiobarbituric acid reaction. Anal Bichem.

[40] Faure P, Lafond JL, Favier AE (1995). Measurement of plasma sulfhydryl and carbonyl groups as a possible indicator of protein oxidation. Analysis of free radicals in biological systems.

[41] Morimoto Y, Shimohara K, Oshima S, Sukamoto T (1991). Effects of the new anti-ulcer agent KB-5492 on experimental gastric mucosal lesion and gastric mucosal defensive factors, as compared to those of teprenone and cimetidine. Japan J Pharmacol.

[42] Arimura A, Lundqvist G, Rothman J, Chang R, Fernandez-Durango R, Elde R (1978). Radioimmunoassay of somatostatin. Metabolism.

[43] Slingerland DW, Cardarelli JA, Burrows BA, Miller A (1984). The utilty of serum gastrin levels in assessing the significence of low serum B12 levels. Arch Intern Med.

[44] Hiruma-Lima CA, Calvo TR, Rodrigues CM, Andrade FD, Vilegas W, Brito AR (2006). Antiulcerogenic activity of *Alchornea castaneaefolia*: effects on somatostatin, gastrin and prostaglandin. J Ethnopharmacol.

[45] Bonamin F, Moraes TM, Kushima H, Silva MA, Rozza AL, Pellizzon CH (2011). Can a *Strychnos* species be used as antiulcer agent? Ulcer healing action from alkaloid fraction of *Strychnos pseudoquina* St. Hil. (Loganiaceae). J Ethnopharmacol.

[46] Luiz-Ferreira A, Cola M, Barbastefano V, De-Faria FM, Almeida AB, Farias-Silva E (2012). Healing, Antioxidant and Cytoprotective Properties of *Indigofera truxillensis* in Different Models of Gastric Ulcer in Rats. Int J Mol Sci.

[47] Okabe S, Amagase K (2005). An overview of acetic acid ulcer models--the history and state of the art of peptic ulcer research. Biol Pharm Bull.

[48] Wallace JL, Devchand PR (2005). Emerging roles for COX-2 in gastrointestinal mucosal defense-review. Br J Pharmacol.

[49] Tarnawski A (2005). Cellular and molecular mechanisms of gastrointestinal ulcer healing. Digestion and Disease Science.

[50] Tarnawski A, Douglass TG, Stachura J, Krause WJ (1991). Quality of gastric ulcer healing: histological and ultrastructural assessment. Alimentary Pharmacology and Therapeutic.

[51] Ito M, Segami T, Inagguma K, Suzuki Y (1994). Cimetidine and omeprazole accelerate gastric ulcer healing by an increase in gastrin secretion. Eur J Pharmacol.

[52] Shirin H, Pinto JT, Liu LU, Merzianu M, Sordillo EM, Moss SF (2001). *Helicobacter pylori* decreases gastric mucosal glutathione. Cancer Lett.

[53] Avila JR, de la Lastra CA, Martin MJ, Motilva V, Luque I, Delgado D (1996). Role of endogenous sulphydryls and neutrophil infiltration in the pathogenesis of gastric mucosal injury induced by piroxicam in rats. Inflamm Res.

[54] Wallace JL, Miller MJ (2000). Nitric oxide in mucosal defense: A little goes a long way. Gastroenterology.

[55] Chandranath SI, Bastaki SM, Singh J (2002). A comparative study on the activity of lansoprazole, omeprazole and PD-136450 on acidified ethanol and indomethacin-induced gastric lesions in the rat. Clinical Experimental Pharmacology and Physiology.

[56] Li Y, Zhang JF, Zhang YM, Ma XB (2009). The protective effect of genistein post conditioning on hypoxia/reoxygenation-induced injury in human gastric epithelial cells. Acta Pharmacol.

[57] Andrews FJ, Malcontenti C, O’Brien PE (1992). Sequence of gastric mucosal injury following ischemia and reperfusion. Role of reactive oxygen metabolites. Dig Dis Sci.

[58] Wada K, Kamisaki Y, Ohkura T, Kanda G, Nakamoto K, Kishimoto Y (1998). Direct measurement of nitric oxide release in gastric mucosa during ischemia–reperfusion in rats. Am J Physiol.

[59] Chamoun F, Burne M, O’Donnell M, Rabb H (2000). Pathophysiologic role of selectins and their ligands in ischemia/reperfusion injury. Front Biosci.

[60] Piper HM, Meuter K, Schäfer C (2003). Cellular mechanisms of ischemia-reperfusion injury. Ann Thorac Surg.

[61] Rao CV, Vijayakumar M (2007). Protective effect of (+)-catechin against gastric mucosal injury induced by ischaemia-reperfusion in rats. J Pharm Pharmacol.

[62] Smith GS, Mercer DW, Cross JM, Barreto JC, Miller TA (1996). Gastric injury induced by ethanol and ischemia-reperfusion in the rat. Differing roles for lipid peroxidation and oxygen radicals. Dig Dis Sci.

[63] Makoto S, Takashi J (2007). Oxidative stress and ischemia–reperfusion injury in gastrointestinal tract and antioxidante, protective agents. J Clin Biochem Nutr.

[64] Brzozowski T (2006). Prostaglandin/cyclooxygenase pathway in ghrelin-induced gastroprotection against ischemia–reperfusion injury. Pharmacol Exp Ther.

[65] Sener G, Sert G, Ozer Sehirli A, Arbak S, Uslu B, Gedik N (2006). Pressure ulcer-induced oxidative organ injury is ameliorated by beta-glucan treatment in rats. Int Immunopharmacol.

[66] Karmeli F, Eliakim R, Okon E, Rachmilewitz D (1994). Gastric and mucosal damage by ethanol is mediated by substance P and prevented by ketotifen, a mast cell stabilizer. Gastroenterology.

[67] Sun FP, Song YG, Cheng W, Zhao T, Yao YL (2002). Gastrin, somatostatin, G and D cells of gastric ulcer in rats. World J Gastroenterol.

[68] Konturek SJ, Radecki T, Brzozowski T, Drozdowicz D, Piastucki I, Muramatsu M (1996). Antiulcer and gastroprotective effects of solon, a synthetic flavonoid derivative of sophoradin- Role of endogenous prostaglandins. Eur J Pharmacol.

[69] Kutchai HC, Berne RM, Levy MN (1996). Gastrointestinal secretions. Principles of Physiology.

